# Silver split nano-tube array as a meta-atomic monolayer for high-reflection band

**DOI:** 10.1038/s41598-022-17703-0

**Published:** 2022-08-10

**Authors:** Yi-Jun Jen, Po-Chun Lin, Xing-Hao Lo

**Affiliations:** Department of Electro-Optical Engineering, National Taipei University of Technology, Taipei, 106 Taiwan

**Keywords:** Nanoscience and technology, Optics and photonics

## Abstract

In this work, an ultra-thin silver film-coated grating as a split silver nanotube array exhibits not only high TE polarized reflectance as a conventional subwavelength grating but also high TM polarized reflectance that is close to or higher than TE reflectance at certain wavelength range. The TM reflectance peak shifts with the morphology of the silver covering. The near-field analysis reveals that the silver nanotube array is an ultra-thin optical double negative metamaterial. The negative permeability associated magnetic field reversal is induced within the grating that is surrounded by a split current loop at the TM reflectance peak wavelength. The near field simulation is used to retrieve the equivalent electromagnetic parameters and optical constants that cause the anomalous TM high reflection. It is demonstrated that the TM impedances have a low magnitude and high magnitude with respect to unity for light incident onto the top and bottom of the grating at the peak wavelength, respectively.

## Introduction

High reflectivity is the optical feature of noble metal elements such as silver, gold, and aluminum. Electrons in metals are only loosely attached to the metal atoms, so they can move under the influence of external electric field. When light hits the metal, the electrons interact with the light and cause it to reflect^1,2^. The relative permittivity of a typical metal usually has a negative real part which magnitude is large compared with unity (the permittivity of air)^3^. The associated normalized impedance, the reciprocal of refractive index, has a small real part compared with unity (the normalized impedance of air) for high reflection at surface of metal. However, according to the well-known reflection coefficient between air and any medium, a medium could exhibit high reflection with impedance magnitude much larger than unity. Such material with large real or imaginary part of impedance does not exist in nature, so a metamaterial with tailored permeability and permittivity can be developed as a high impedance medium. It can be estimated that the phase of a reflected wave from a medium surface can be engineered by tailoring a complex impedance for high reflection.

Various subwavelength nanostructures as electric or magnetic resonators have been developed as artificial atoms for metamaterials. The tailorable permittivity and permeability of metamaterial realize extraordinary optical property through the tailored optical constants including refractive index and impedance^4^. Typical anomalous optical properties of metamaterials with negative indexes of refraction have been observed from matrixes or arrays of split rings^5,6^, metal nanorods^7–9^, and fishnet structure^10,11^. The metamaterials enable us to realize optical cloaking, develop a superlens that can overcome diffraction^12–15^ and highly sensitive sensors^16–20^. Following advances in nanofabrication, a negative-index medium can be designed and fabricated for microwave to visible wavelengths^21^. Recently, periodically distributed ultrathin nanostructures with a subwavelength scale on a surface have been be designed as metasurfaces^22^, supporting unprecedented control of light propagation, including anomalous reflection and refraction^23–25^, manipulation of the polarization state of electromagnetic waves^26,27^, and polarization-dependent steering of light beams^28,29^. This control has applications in ultrathin flat lenses^30–32^, perfect absorbers^33^, polarization detectors^34^, waveplates and other devices^35^. The anomalous reflection and refraction rely on periodically arranged supercells that provide a phase shift of 2π on a surface*,* consistent with a generalized form of Snell’s law. The basic element of a supercell is a dielectric or metallic nanostructure, which interacts with an electromagnetic wave to generate a phase change for transmitted or reflected wave.

The element of a metasurface could be an artificial atom of optical metamaterial which property can be understood from an atomic layer of metamaterial. Typical optical metamaterials comprise artificial subwavelength atoms with tailorable electric or magnetic dipole moments. These artificial atoms can be elements of a metasurface and the role of each element in manipulating light wave can be understood from a regularly distributed mono-atomic layer of metamaterial which tailorable equivalent impedance and refractive index will give great flexibility in design and fabrication of a metasurface.

Recently, a silver coated nanopillar array was developed as a metasurface (meta-mirror) for ultrahigh resolution organic light-emitting diode. The meta-mirror reflected light wave with reflection phase that could be altered through varying the pitch and high of nanopillars^36^. However, the elements of the metasurface was not shown to be artificial atoms that lead to extraordinary permittivity and permittivity.

In this work, an atomic metamaterial layer with double negative indexes (both permittivity and permeability are negative) is developed as a meta-mirror at certain wavelengths. A nanoimprinted dielectric grating on whose ridges is obliquely coated an ultra-thin silver film is formed as a silver split nano-tube array (SNTA). The cross-sectional shape of each split nano-tube is similar to the shape of a split ring^6^. Generally, a subwavelength metal grating transmits the transverse magnetic (TM) polarized wave whose oscillating electric field is perpendicular to the grating and reflects the transverse electric (TE) polarized wave whose oscillating electric field is parallel to the grating^37,38^. However, a strong TM reflectance occurred at certain wavelengths and the high TM reflection peak was shifted from visible to infrared wavelength range by varying the morphology of covered silver. A near-field simulation revealed that at resonant frequencies, enhanced reversal of the magnetic field was induced within the split nano-tube and an abnormal TM polarized high reflection occurred. The equivalent optical constants including impedance and refractive index of the array were obtained from the reflection and transmission coefficients that were themselves obtained by treating the layer as a bianisotropic metamaterial^39^. Then the equivalent electromagnetic parameters including permittivity and permeability were retrieved from the impedance and refractive index. The retrieved permeability was negative real for TM polarization and in agree with the phenomenon of localized magnetic field reversal. It is demonstrated that the TE and TM high reflection came from different mechanisms. The TE impedance is similar to a metal with low magnitude compared with unity. However, the TM impedance is strongly depedent on the direction of light propogation.

## Results and discussion

**Figure 1:**
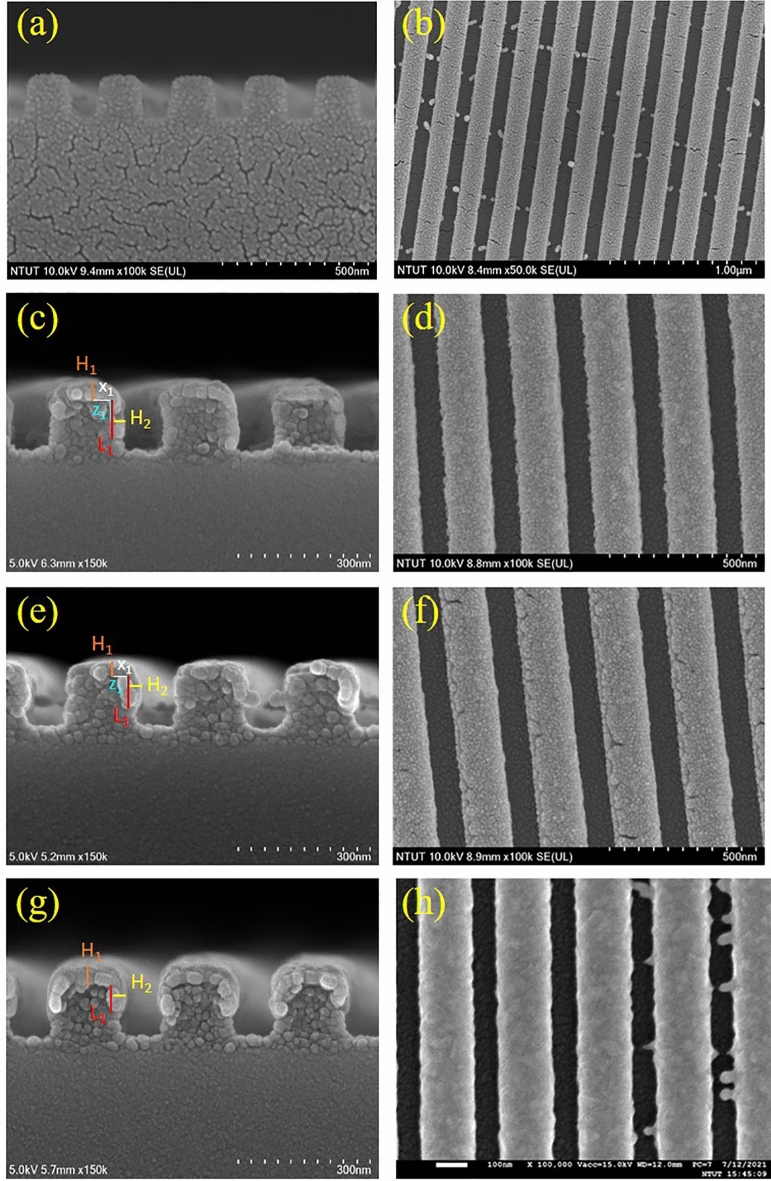
Scanning electron microscopy (SEM) images of fabricated SNTAs. (**a**) Cross-sectional and (**b**) top-view images of bare grating. (**c**) Cross-sectional and (**d**) top-view images of SNTA-1. (**e**) Cross-sectional and (**f**) top-view images of SNTA-2. (**g**) Cross-sectional and (**h**) top-view images of SNTA-3.

**Figure 2:**
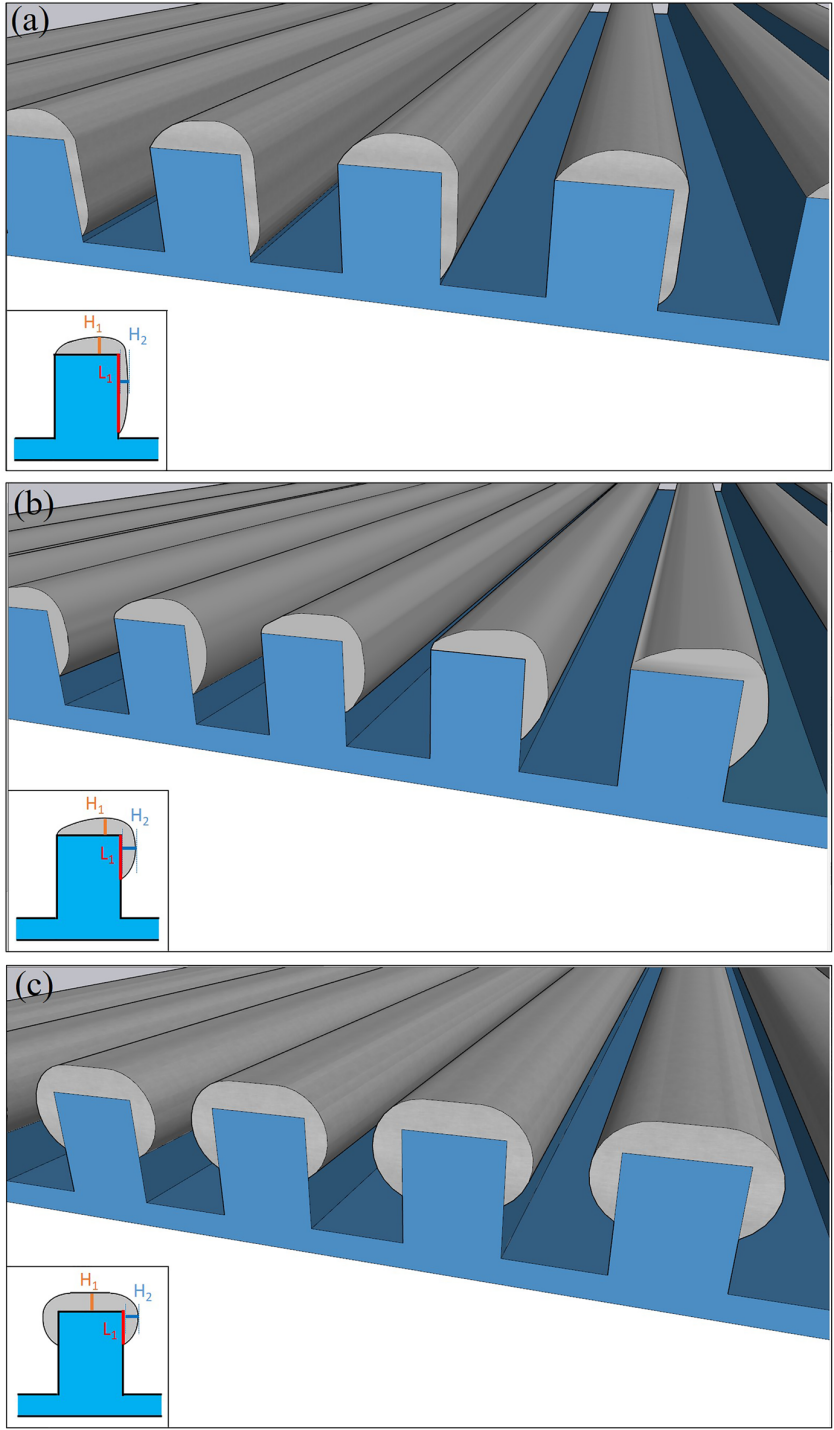
Schematic-drawings of silver coated on gratings: (**a**) SNTA-1, (**b**) SNTA-2, (**c**) SNTA-3.

The bare dielectric gratings were purchased from Ever Radiant Inc. Taiwan. They were fabricated using a hot embossing system to form a Poly(diallyl phthalate) (PDAP) pattern on a BK7 substrate. The surface was patterned with a periodic nanograting with a pitch of 250 nm, a height of 160 nm and a width of 125 nm. Nanostructured PDAP was used as a substrate for the oblique deposition of silver. Silver was deposited on the PDAP grating in an electron beam evaporation system. (Detailed deposition method was performed in the Experimental Methods.) The grating was tilted at a deposition angle of ${\uptheta }_{v}$ between the normal to the substrate holder and the deposition flux. A quartz monitor was mounted next to the rotational stage, allowing the amount of deposited silver to be controlled using a quartz mass monitor adjacent to the substrate holder. The deposition rate, controlled by the quartz monitor, was 0.3 nm/s. A monitor ensured that a 20 nm-thick layer of silver was deposited onto each PDAP grating. The gratings on which silver was deposited at a ${\uptheta }_{v}$ of 40° and 50° are denoted as SNTA-1 and SNTA-2, respectively. The bare grating was bi-deposited obliquely by depositing silver firstly at ${\uptheta }_{v}$ = 50° and then at ${\uptheta }_{v}$ = − 50° (see Supplementary Fig. 1). The deposited amount of silver was also thickness of 20 nm on the monitor at each angle. The bi-deposited sample is denoted as SNTA-3. Figure 1 presents top-view and cross-sectional SEM images of the bare grating and the three samples. For the SNTA-1 and SNTA-2, silver was obliquely deposited on each grating’s top side and lateral side facing the evaporation source. Due to the oblique deposition, the thickest positions (x_1_ and z_1_) on the top and lateral sides are located near the edge of grating: (x_1_, z_1_) are (45 nm, 55 nm) and (35 nm, 20 nm) for the SNTA-1 and SNTA-2, respectively. The thicknesses at x_1_ and y_1_ are denoted as H_1_ and H_2_, respectively. Owing to the shadowing effect, the lower deposition angle caused deeper growth of the silver on the lateral side. From the SEM image of SNTA-3, the coated silver is symmetrical to the center of grating and the thickness on the top and both lateral sides is approximately 39 nm. For SNTA-1, it is obvious that the thickness of coated silver on the lateral side is thinner than the thickness of silver on the top. Table 1 shows the morphology parameters that were observed from the SEM images. The morphology of the capped silver on the grating was schematically specified in terms of depth L_1_ and thickness H_1_ on the top and thickness H_2_ on the lateral side of the PDAP, as shown in Fig. 2. According to the aforementioned estimation, the top thickness H_1_ of SNTA-1 exceeded that of SNTA-2 but the lateral thickness H_1_ of SNTA-1 was less than that of SNTA-2. The L_1_ of SNTA-1 exceeded that of SNTA-2. The morphology parameters will be tuned to have the simulated reflectance and transmittance spectra in agreement with measurement results.

**Table 1:** The observed morphology parameters of SNTA-1, SNTA-2, and SNTA-3 from SEM images.

Sample	L_1_ (nm)	H_1_ (nm)	H_2_ (nm)
SNTA-1	111 ± 16	39 ± 4	27 ± 4
SNTA-2	86 ± 8	33 ± 2	30 ± 2
SNTA-3	71 ± 9	39 ± 3	32 ± 2

With the plane of incidence perpendicular to the grating, the transverse magnetic (TM) mode and transverse electric (TE) mode reflectance (R) and transmittance (T) of each sample from wavelengths of $\uplambda =380 \, \mathrm{ nm}$ to $\uplambda =2000 \, \mathrm{ nm}$, were measured using a spectrometer (UH4150, Hitachi). The extinctance (E) was calculated using the relation E = 1-R-T. The TE reflectance (R_TE_) spectra of SNTA-1 and SNTA-2 are similar. For SNTA-1, R_TE_ increases from 20.4% at $\uplambda$ = 380 nm to 93.9% at $\uplambda$ = 2000 nm. For SNTA-2, R_TE_ increases from 16.5% at $\uplambda$ = 380 nm to 91.0% at $\uplambda$ = 2000 nm. Since the silver layer on the top of SNTA-3 is thicker than the others, the TE reflectance exceeds 90.0% from 638 to 2000 nm, exceeding those of the other two samples. However, the TM reflectance spectrum includes major reflection peaks at wavelengths of 971 nm, 855 nm, and 1005 nm, with maximum reflectance values of 61.8%, 74.2%, and 91.9% for SNTA-1, SNTA-2, and SNTA-3, respectively. The major reflectance peaks accompany transmittance dips with minimum values of 5.6%, 2.2%, and 0.4% at wavelengths of 961 nm, 852 nm, and 1005 nm for SNTA-1, SNTA-2, and SNTA-3, respectively. For SNTA-3, the extinctance value is 7.7% and smallest compared with the other two samples at the peak wavelength. For SNTA-2, the maximum TM reflectance (R_TM_) is 74.2%, which exceeds the R_TE_ of 62.9% at $\uplambda$ = 855 nm. For SNTA-3, both the maximum R_TM_ of 91.9% and the R_TE_ of 97.0% at $\uplambda$ = 1005 nm exceed 90%. A minor TM reflectance peak with a maximum R_TM_ of 47.9% was observed at $\uplambda$ = 440 nm for SNTA-3. At the same wavelength, a TM transmittance dip with minimum transmittance of 18.5% and maximum extinctance of 33.6% were measured.

**Figure 3:**
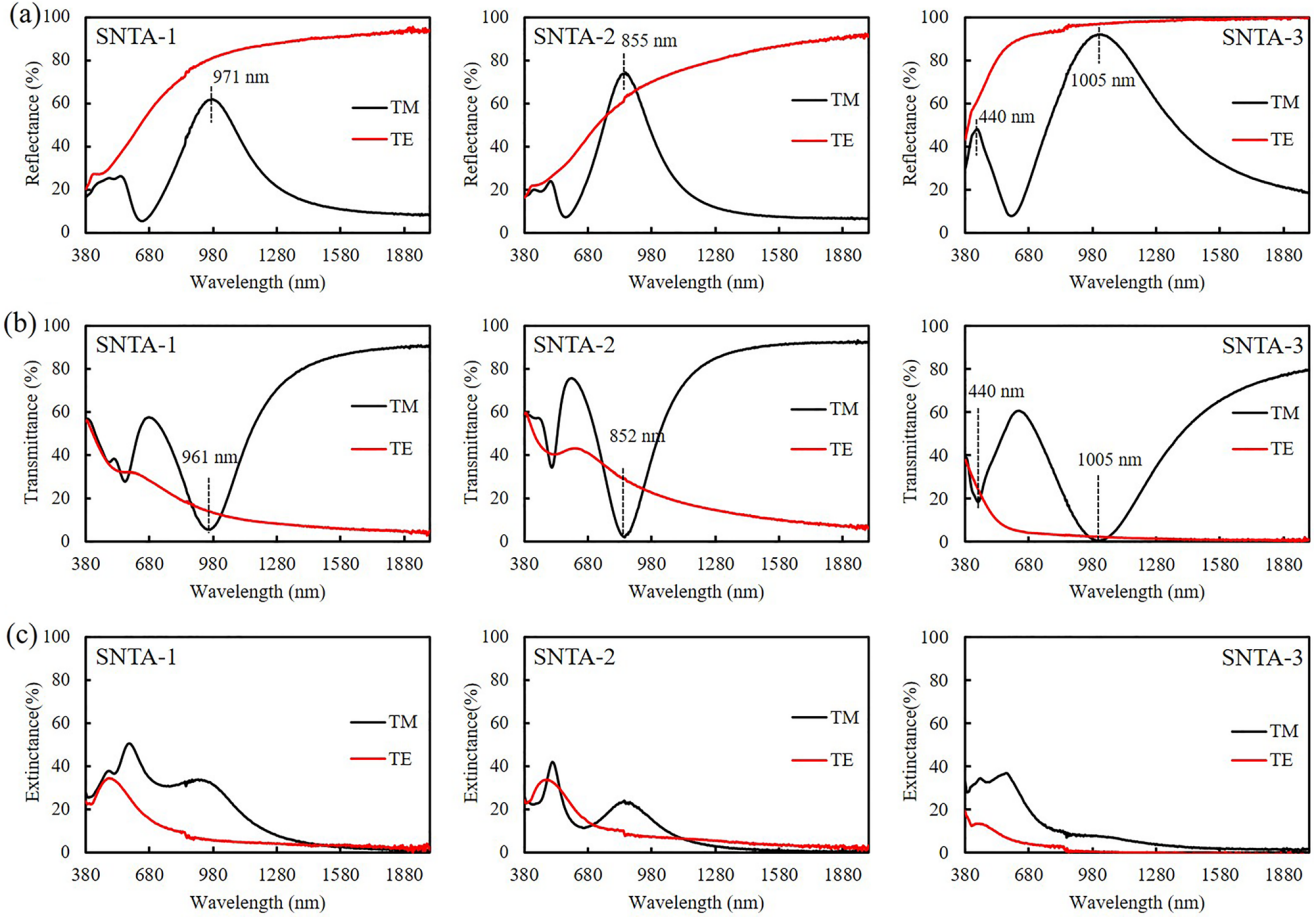
TM and TE polarized spectra of (**a**) reflectance (R), (**b**) transmittance (T) and (**c**) extinctance (E) of each sample.

**Figure 4:**
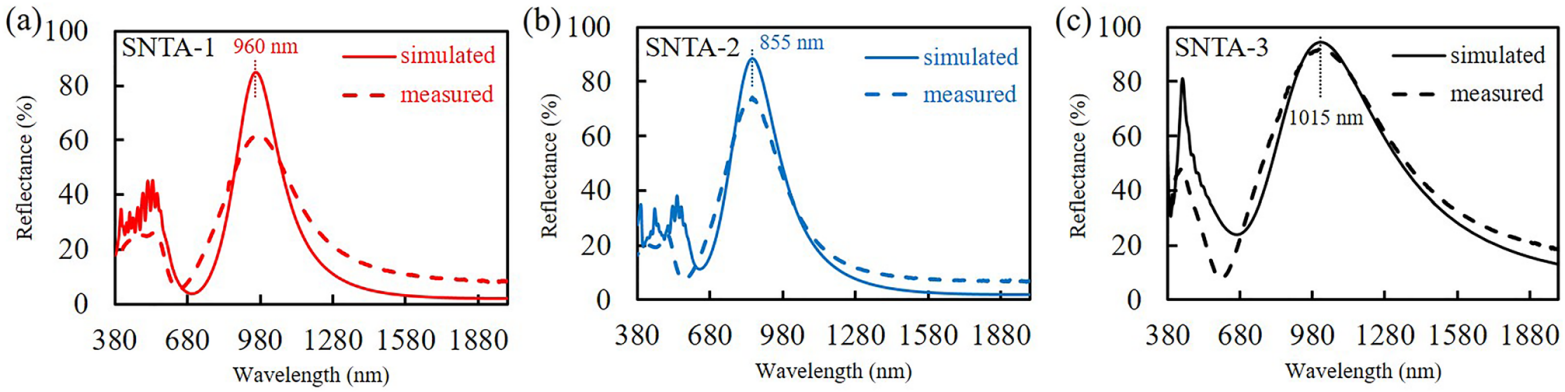
Simulated and measured R_TM_ spectra of (**a**) SNTA-1, (**b**) SNTA-2, (**c**) SNTA-3.

In order to elucidate the mechanism that causes the TM reflection peak, a finite- difference time-domain (FDTD) simulation (Lumerical Inc.) was performed to observe the near-field distributions of electric field and magnetic field. In the simulation, a harmonic TM plane wave with amplitude of unity at the normal incidence was used and the size of the mesh was fixed at 5 nm. The morphology parameters in Fig. 2 were adopted and then revised to simulate the reflectance and transmittance spectra of the Air/grating/BK7 glass system. The refractive index of silver was taken from the database of Macleod Package^40^. The refractive index of PDAP (see Supplementary Fig. 2) was measured using an ellipsometer (J. A. Woollam Co., M2000). The morphologies of all samples were modified by tuning the parameters of H_1_, H_2_, and L_1_ so that the simulated TM reflectance major peaks were quite close to the measured results in Fig. 3. Figure 4 shows the simulated TM reflectance spectra compared with measured spectra. The morphology parameters of the silver on the gratings in the simulation are listed in Table 2. The cover thicknesses H_1_ of SNTA-1, SNTA-2, and SNTA-3 are 41 nm, 30 nm, and 42 nm, respectively. The depth L_1_ = 140 nm of SNTA-1 is indeed larger than that of SNTA-2 (L_1_ = 88 nm) as aforementioned estimation.

**Table 2:** The retrieved morphology parameters of SNTA-1, SNTA-2, and SNTA-3 from simulation.

Sample	L_1_ (nm)	H_1_ (nm)	H_2_ (nm)
SNTA-1	140	41	18
SNTA-2	88	30	29
SNTA-3	72	42	30

**Figure 5:**
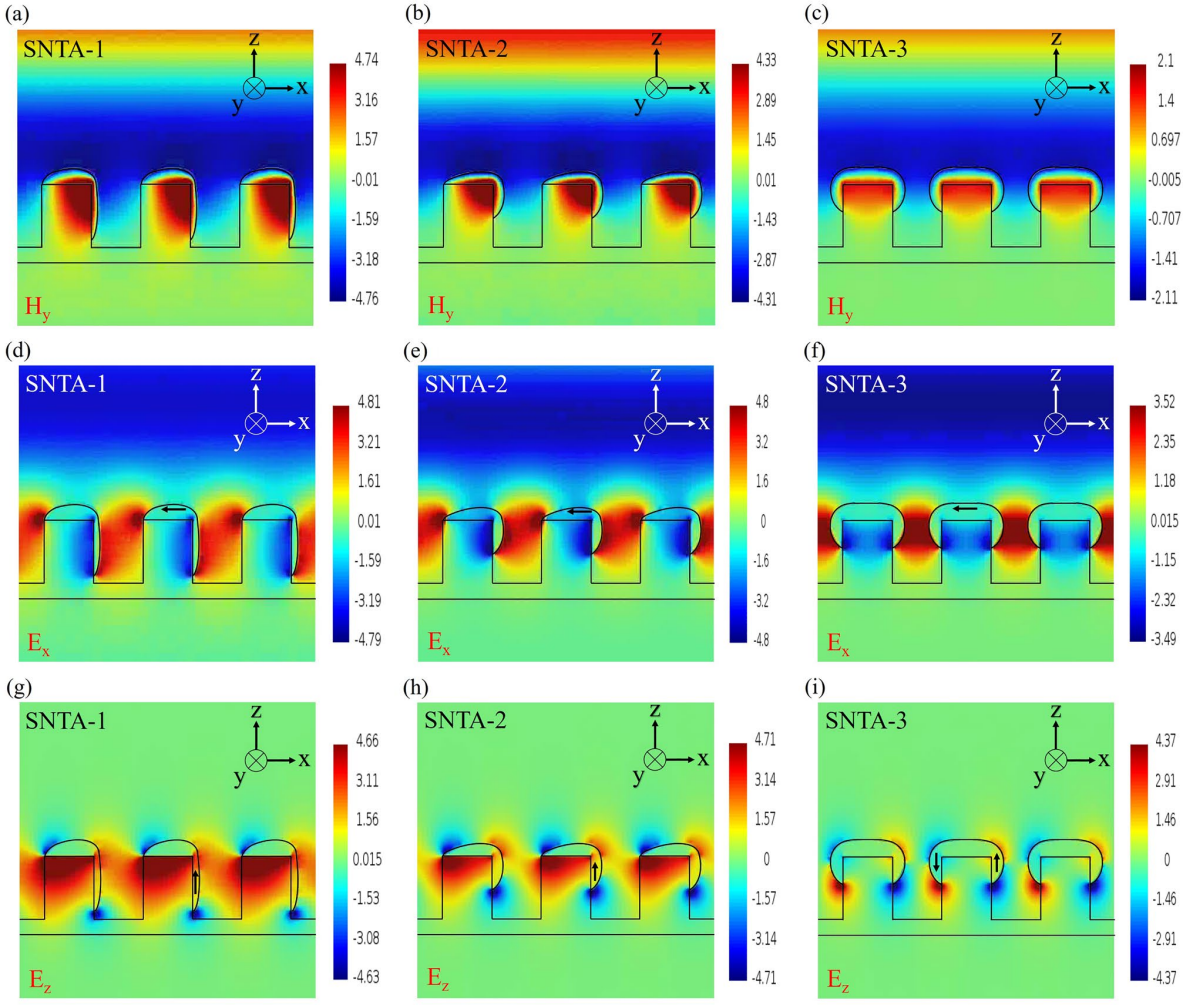
Instantaneously captured images of the oscillating (**a**)–(**c**) magnetic field, (**d**)–(**f**) electric field component E_x_, and (**g**)–(**i**) electric field component E_z_ for each sample.

Figure 5 plots instantaneously images of the oscillating magnetic field and electric field at peak wavelengths of 971 nm, 855 nm, and 1005 nm for SNTA-1, SNTA-2, and SNTA-3, respectively. The electromagnetic wave was applied to normally propagate through each SNTA and the amplitude of electric field is unitary with units of V/m, and the magnetic field units of A/m. The images in Fig. 5 were captured at the moment corresponding to maximum magnetic field within the PDAP. In Fig. 5a and b, a strong magnetic field that was reversed with respect to the propagating field was enhanced and distributed around the corner that was formed by the coated silver. For the SNTA-3, the enhanced and reversed magnetic field was distributed within the area that was surrounded by the silver, as shown in Fig. 5c. Figure 5d–i shows the instantaneous distributions of electric field components E_x_ and E_z_ for the three samples. The directions of associated currents were judged from the components E_x_ and E_z_ within the capped silver. It was demonstrated that the current flow was in counterclockwise to induce the magnetic fields for each sample, as shown in the Fig. 5.

**Figure 6:**
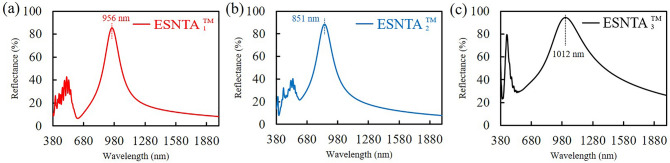
Simulated reflectance spectra of (**a**) ESNTA_1_^TM^, (**b**) ESNTA_2_^TM^, and (**c)** ESNTA_3_^TM^.

**Figure 7:**
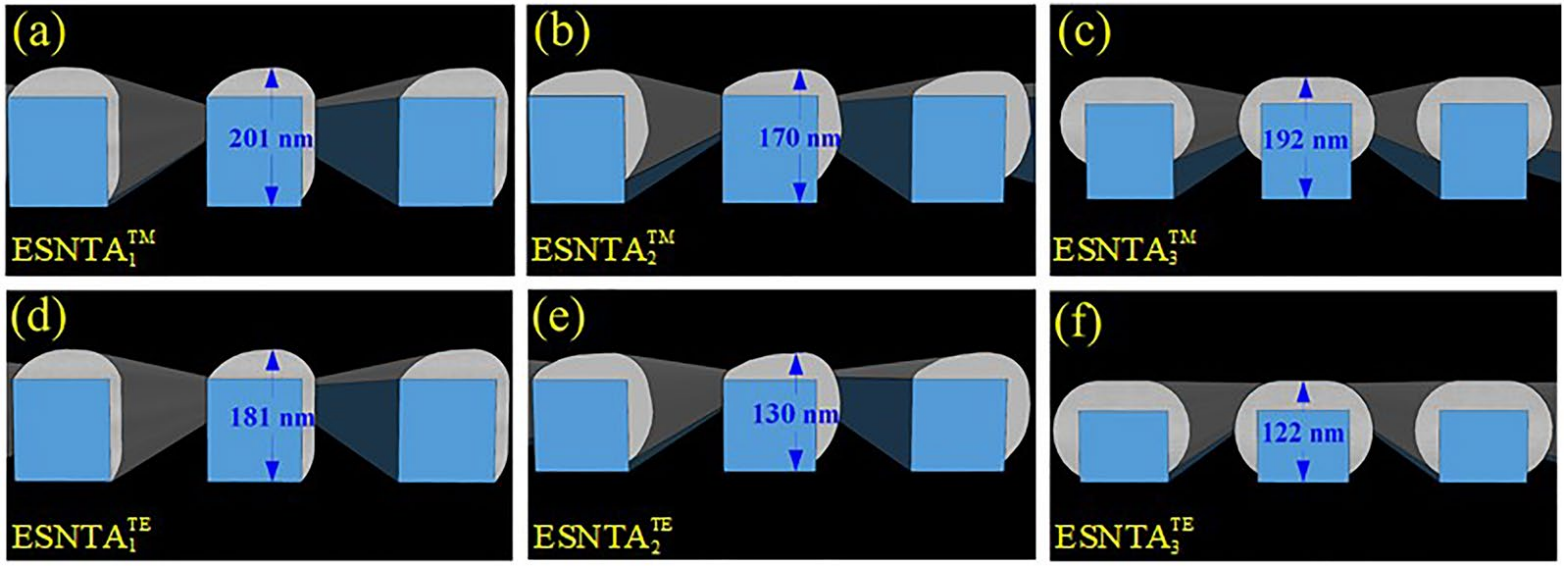
Schematic-drawings of (**a**) ESNTA_1_^TM^, (**b**) ESNTA_2_^TM^, (**c**) ESNTA_3_^TM^, (**d**) ESNTA_1_^TE^, (**e**) ESNTA_2_^TE^, and (**f**) ESNTA_3_^TE^ in free space.

**Figure 8:**
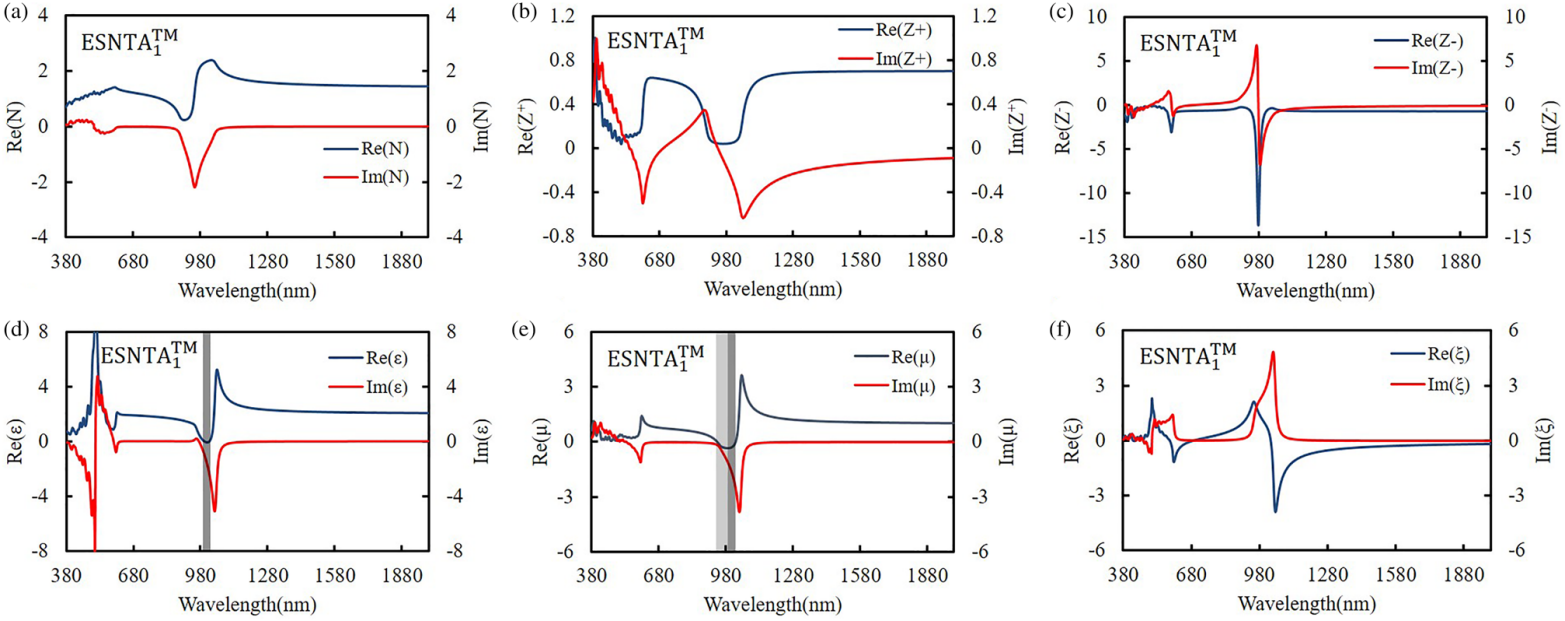
Retrieved (**a**) refractive index, (**b**) positive impedance, (**c**) negative impedance, (**d**) permittivity, (**e**) permeability, and (**f**) bianisotropy parameter for ESNTA_1_^TM^.

**Figure 9:**
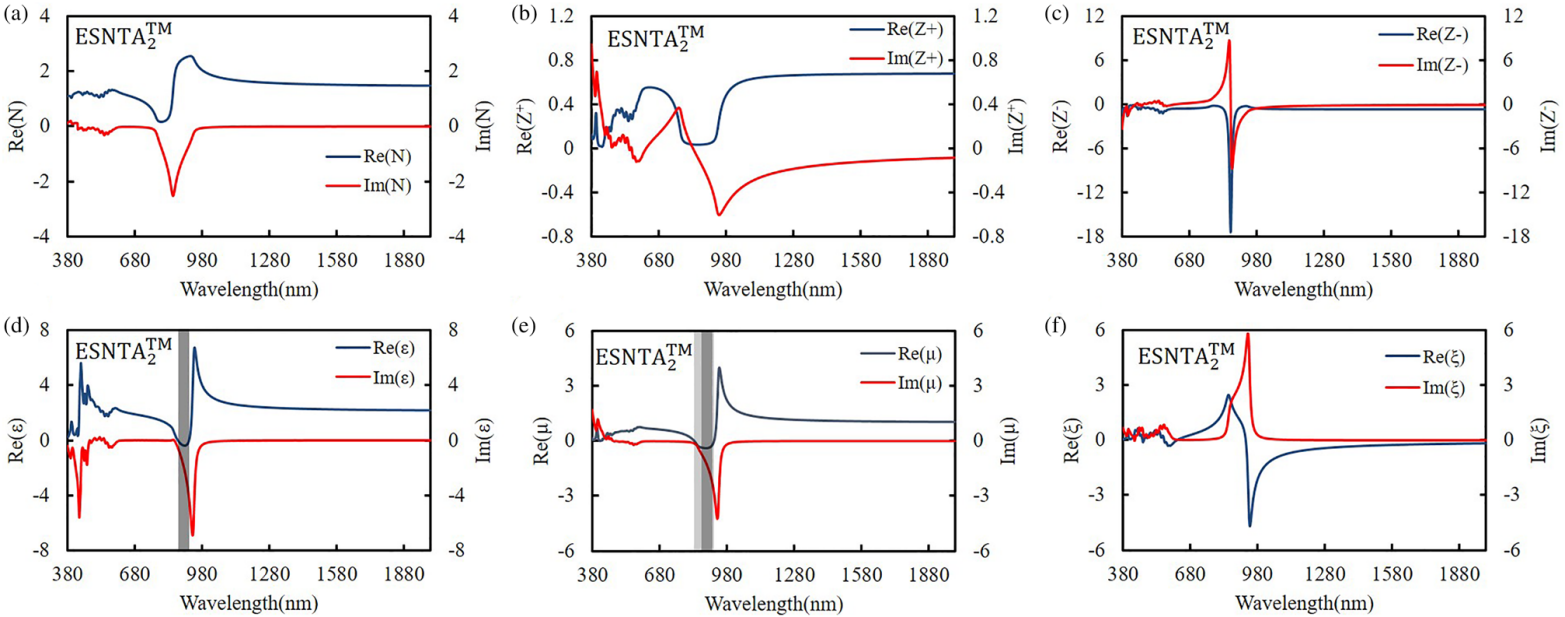
Retrieved (**a**) refractive index, (**b**) positive impedance, (**c**) negative impedance, (**d**) permittivity, (**e**) permeability, and (**f**) bianisotropy parameter for ESNTA_2_^TM^.

**Figure 10:**
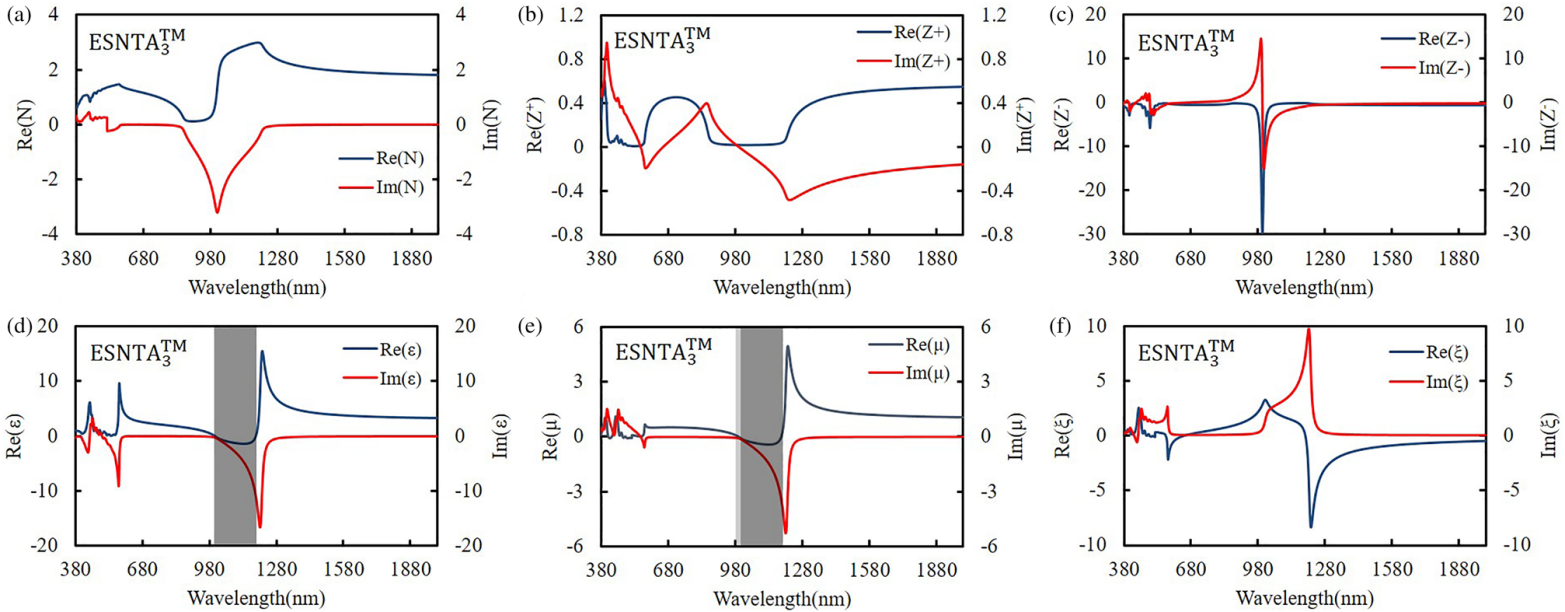
Retrieved (**a**) refractive index, (**b**) positive impedance, (**c**) negative impedance, (**d**) permittivity, (**e**) permeability, and (**f**) bianisotropy parameter for ESNTA_3_^TM^.

**Figure 11:**
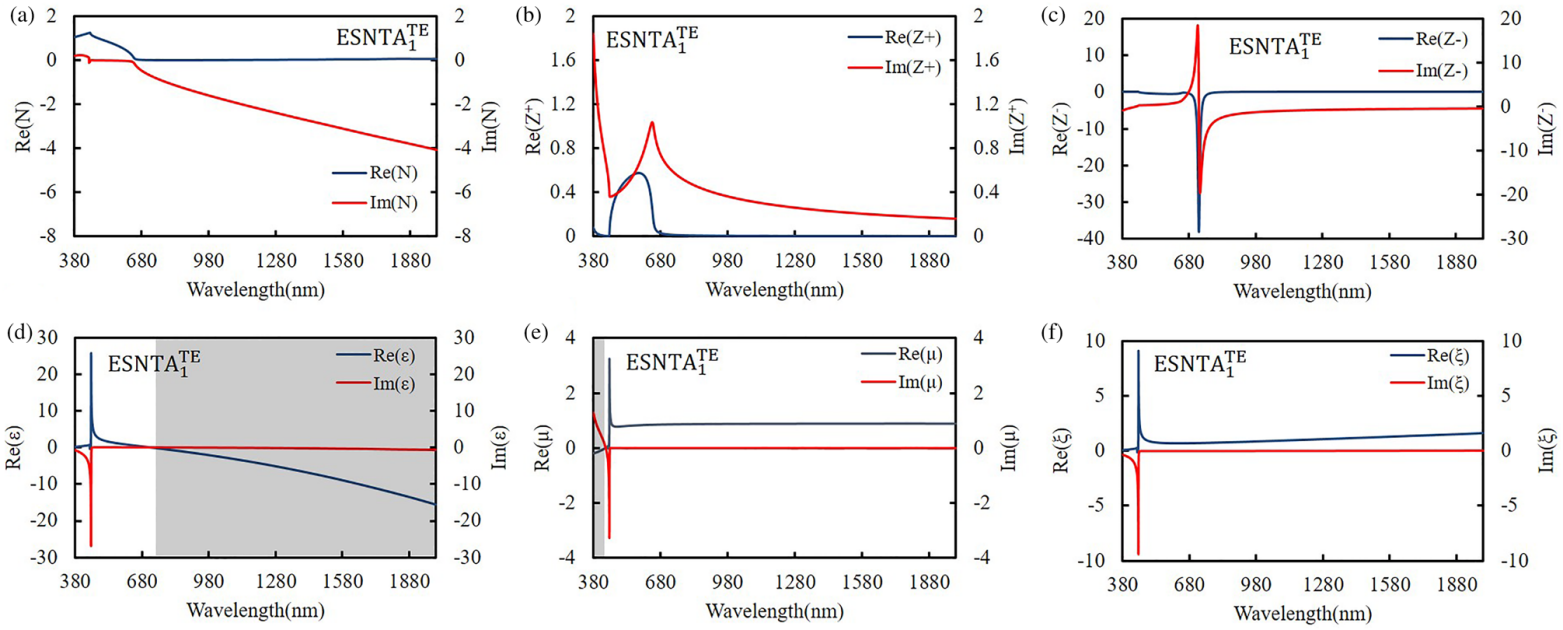
Retrieved (**a**) refractive index, (**b**) positive impedance, (**c**) negative impedance, (**d**) permittivity, (**e**) permeability, and (**f**) bianisotropy parameter for ESNTA_1_^TE^.

**Figure 12:**
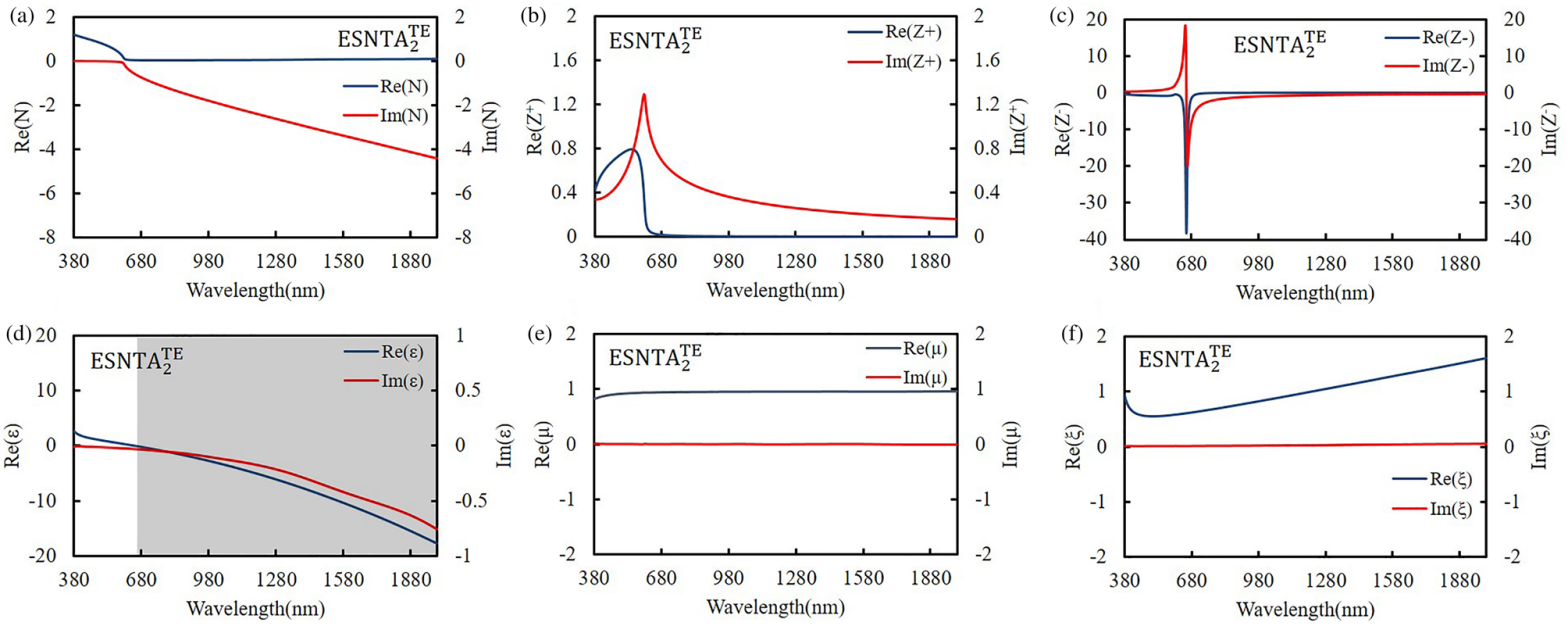
Retrieved (**a**) refractive index, (**b**) positive impedance, (**c**) negative impedance, (**d**) permittivity, (**e**) permeability, and (**f**) bianisotropy parameter for ESNTA_2_^TE^.

**Figure 13:**
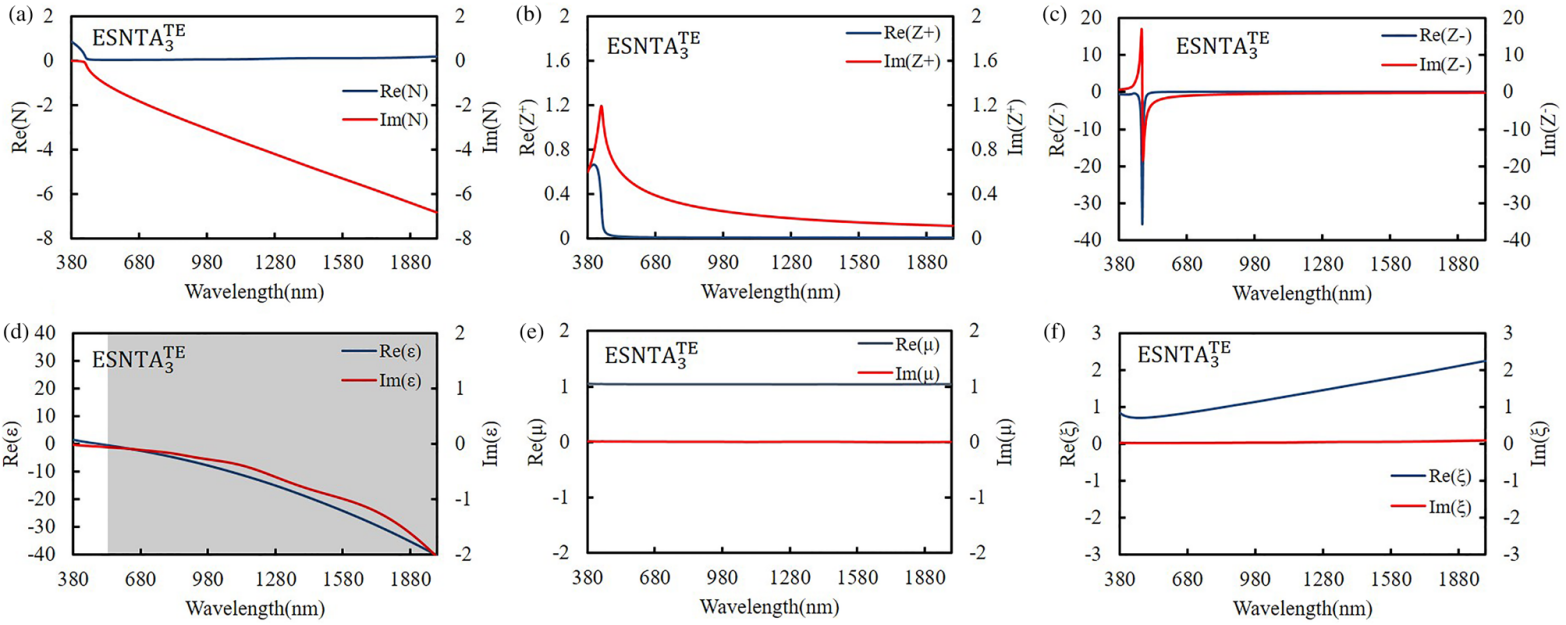
Retrieved (**a**) refractive index, (**b**) positive impedance, (**c**) negative impedance, (d) permittivity, (**e**) permeability, and (**f**) bianisotropy parameter for ESNTA_3_^TE^.

The silver-covered grating was extracted without substrate and the grating bottom as a silver split nanotube array. When the grating was illuminating with an electromagnetic wave, the local field enhancement only exists at top of each ridge. The induced magnetic dipole moment decided the size of meta-atom, so the mono meta-atomic monolayer needed to be extracted from the whole grating structure. The extracted layer was set to keep the spectra as measured result and contain local field distribution within it. The thickness of extracted layer was determined from the top of silver to a minimum depth without shifting the major TM reflectance peak more than 5 nm from the simulation peak shown in Fig. 4. The split nanotube array was suspended in free space and assumed to be a bianisotropic metamaterial layer for simulation and the reflectance spectrum was simulated and shown in Fig. 6. The thicknesses of the three extracted split nanotube arrays (ESNTA) are 201 nm, 170 nm, and 192 nm for ESNTA_1_^TM^, ESNTA_2_^TM^ and ESNTA_3_^TM^ corresponding to the aforementioned SNTA-1, SNTA-2, and SNTA-3, respectively. The major TM reflectance peak wavelengths and reflectance maxima were (956 nm, 85.4%), (851 nm, 88.7%) and (1012 nm, 94.3%) for ESNTA_1_^TM^, ESNTA_2_^TM^ and ESNTA_3_^TM^, respectively. The thicknesses used for simulation included the magnetic field resonance areas within the gratings shown in Fig. 5. The equivalent optical constants are refractive index N, equivalent impedance Z^+^ for a wave that propagates in the positive direction (toward the silver coated side of grating) and equivalent impedance Z^−^ for a wave that propagates in the negative direction (toward uncoated side of ESNTA), as shown in Fig. 7. The time-dependence e^iωt^ of electromagnetic wave is used with ω as the angular frequency and t as time^9^. Therefore, the imaginary part of the equivalent refractive index N should be negative. N, Z^+^ and Z^−^ are obtained from the reflection and transmission coefficients that were measured for a light wave incident in the positive and negative directions (see Supplementary Fig. 4). The equivalent electromagnetic parameters of relative permittivity $\upvarepsilon$, permeability $\upmu$, and the bianisotropy parameter ξ were derived from N, Z^+^ and Z^−^. These parameters were calculated at the peak wavelengths in the TM reflectance spectrum of each sample. The amplitudes and phases of reflection and the transmission coefficients were derived from the near-field simulation. Figures 8, 9 and 10 present the calculated optical and electromagnetic parameters. On the other hand, the same procedure was executed for TE polarization. The ESNTA^TE^ was also suspended in free space for simulation. The thickness of ESNTA^TE^ determined from the top of silver to a minimum depth without changing its TE reflectance in free space from the simulated reflectance in the Air/SNTA/BK7 glass system more than 5% (see Supplementary Fig. 3). The thicknesses of the three extracted split nanotube arrays (ESNTA^TE^) are 181 nm, 130 nm, and 122 nm for ESNTA_1_^TE^, ESNTA_2_^TE^, and ESNTA_3_^TE^, as shown in Fig. 7. The TE reflectance values were 88.5%, 74.8%, and 97.1% at 956 nm, 851 nm, and 1012 nm for ESNTA-1, ESNTA-2, and ESNTA-3, respectively. Accordingly, the thickness of extracted layer is different from that for TM polarization. Supplementary Fig. 5 shows the retrieved reflection and transmission coefficients from simulation. The equivalent optical constants and electromagnetic parameters were also retrieved for TE polarization, as shown in Figs. 11,12, and 13.

The $\upvarepsilon$ and µ represent the excitation of electric dipoles and magnetic dipoles by the light, respectively. The TM-polarized $\upvarepsilon$ spectra show $\upvarepsilon =0$ at the equivalent inductance and capacitance resonant wavelengths at 1018 nm, 922 nm, and 1187 nm for ESNTA_1_^TM^ ESNTA_2_^TM^ and ESNTA_3_^TM^, respectively. The TM-polarized µ spectra show $\upmu =0$ at the inductance and capacitance resonant wavelengths at 1025 nm, 923 nm, and 1185 nm for ESNTA_1_^TM^ ESNTA_2_^TM^ and ESNTA_3_^TM^, respectively. The light gray areas indicate the wavelength ranges for negative real part of $\upvarepsilon$ and negative real part of µ. The maximum magnitudes of the negative real part of permeability are 0.330, 0.395, and 0.419 for ESNTA_1_^TM^ ESNTA_2_^TM^ and ESNTA_3_^TM^, respectively. The three negative real parts are similar because the average magnetic field within the ridges of the three samples are similar. Although the reversal magnetic fields of ESNTA_1_^TM^ and ESNTA_2_^TM^ are stronger than ESNTA_3_^TM^, their distributions are located near the corners surrounded by silver, as shown in Fig. 5. The reversal magnetic field of ESNTA_3_^TM^ is uniformly distributed within the ridges. Both permittivity and permeability are negative real at the wavelength ranges marled with dark gray areas in Figs. 8, 9, 10), so the split nanotube array is a double negative metamaterial. Notably, the real part of N is positive although electric permittivity and magnetic permeability are negative real because it is partly connected to the the bianisotropy parameter. The magnitude of impedance Z^+^ is low but the magnitude of impedance Z^−^ is high at the wavelength of the TM reflection peak. Both Z^+^ and Z^−^ are different but both of them cause high reflection. The real parts of Z^−^ reach minima (largest absolute values) − 13.600 at 979 nm, − 17.394 at 864 nm, and − 29.667 at 1002 nm for ESNTA_1_^TM^ ESNTA_2_^TM^ and ESNTA_3_^TM^, respectively. For ESNTA_3_^TM^, the Z^+^ and Z^−^ are 0.015–0.027i and − 7.809–13.228i at 1012 nm with magnitudes much smaller and larger than unity, respectively. As shown in Figs. 11,12, and 13, the TE polarized refractive indexes and permittivity values are positive real. The refractive indexes and impedances of ESNTA_1_^TE^ and ESNTA_2_^TE^ are similar. For the three samples, the high TE reflection is due to small magnitude of impedance Z^+^ that are similar to a typical reflective metal.

## Conclusion

In conclusion, a dielectric subwavelength grating that was covered with an ultra-thin silver film yields a TM reflection peak at wavelengths that correspond to the morphology of the silver covering. According to our analysis, the high TM polarized reflection comes from a high magnitude of equivalent impedance that is different from that of a high reflective noble metal. The high impedance is attributed to morphology and wavelength dependent permittivity and permeability. When the SNTA is illuminated with a TM polarized electromagnetic wave, a localized reversed magnetic field is induced by a split current loop for a negative real equivalent permeability. Both complex negative real permittivity and permeability cause an equivalent impedance for high reflection. The phase of TM reflection coefficient is various and dependent on the complex impedance (permittivity and permeability) that can be engineered in the future. Therefore, the phase difference between the TE and TM reflection coefficients enables the SNTA to be a meta-mirror to manipulate the polarization state of a reflected electromagnetic wave efficiently. The tunable high reflection band and reflection phase of such meta-mirror can be applied in Fabry–Perot cavity for novel applications in novel polarization dependent filters, laser, and organic light-emitting diode. On the other hand, the meta-atoms that lead to double negative optical property will be designed and distributed on a surface periodically to achieve a metasurface for manipulating extraordinary light wave propagation and biosensing in the future.

## Methods

Oblique angle deposition of silver was evaporated at a rate of 0.3 nm/s in a custom-built physical vapor deposition system. The evaporation was run in a prior background vacuum of 10^−6^–10^−7^ Torr. This process was monitored by a quartz crystal microbalance located near the sample. The distance between the crucible of an electron beam evaporation and the center of substrate is 301 mm. An electron-beam accelerating voltage of 6.0 kV and emission current of 95–100 mA were applied. Silver was deposited at a deposition angle (${\uptheta }_{v}$) of 40° and 50° with respect to the substrate normal. The substrate area was 24 × 25 mm^2^ for deposition. The substrate temperature was maintained at 10 °C during deposition using the water cooling system.

## Supplementary Information

Supplementary Figures.

## Data Availability

All data generated or analysed during this study are included in this published article (and its Supplementary Information files).
