# Supplementary material for: Silver split nano-tube array as a meta-atomic monolayer for high-reflection band
Source: Sci Rep. 2022 Aug 10;12:13611. doi: 10.1038/s41598-022-17703-0 (PMC9365859; doi:10.1038/s41598-022-17703-0)
Supplement: Supplementary file 1 — Supplementary Figures. [file 41598_2022_17703_MOESM1_ESM.pdf]

Supplementary Information for

Silver split nano-tube array as a meta-atomic monolayer for high-reflection band

Yi-Jun Jen [1], Po-Chun Lin[1], Xing-Hao Lo[1]*

Department of Electro-Optical Engineering, National Taipei University of Technology, Taipei 106, Taiwan

*Correspondence: jyjun@ntut.edu.tw

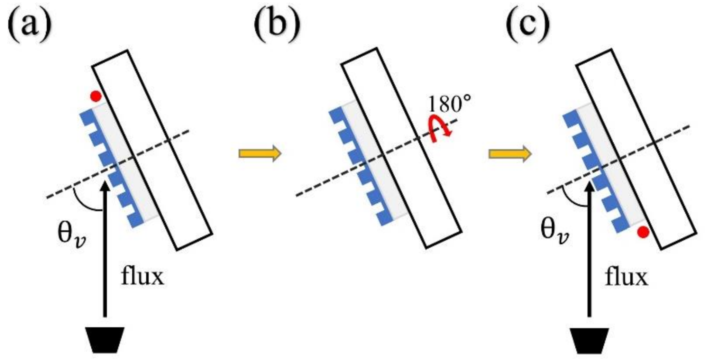

Supplementary Figure 1. Oblique-angle deposition of silver on PDAP grating in an e-beam chamber: (a) oblique deposition at θ; (b) 180° rotation of substrate holder; (c) oblique deposition at –θ

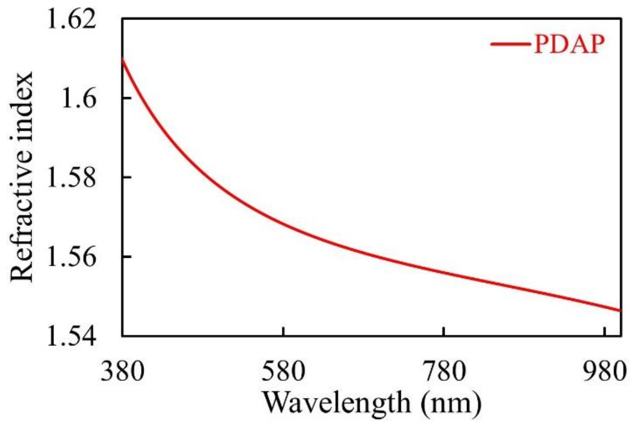

Supplementary Figure 2. Refractive index spectrum of PDAP

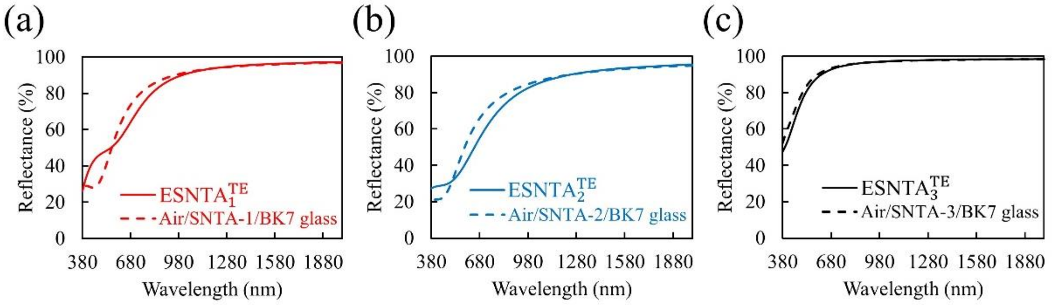

Supplementary Figure 3. Simulated reflectance spectra of (a) ESNTA$_1^{TE}$ in free space and Air/SNTA-1/BK7 glass system, (b) ESNTA$_2^{TE}$ in free space and Air/SNTA-2/BK7 glass system, (c)ESNTA$_3^{TE}$ in free space and Air/SNTA-3/BK7 glass system.

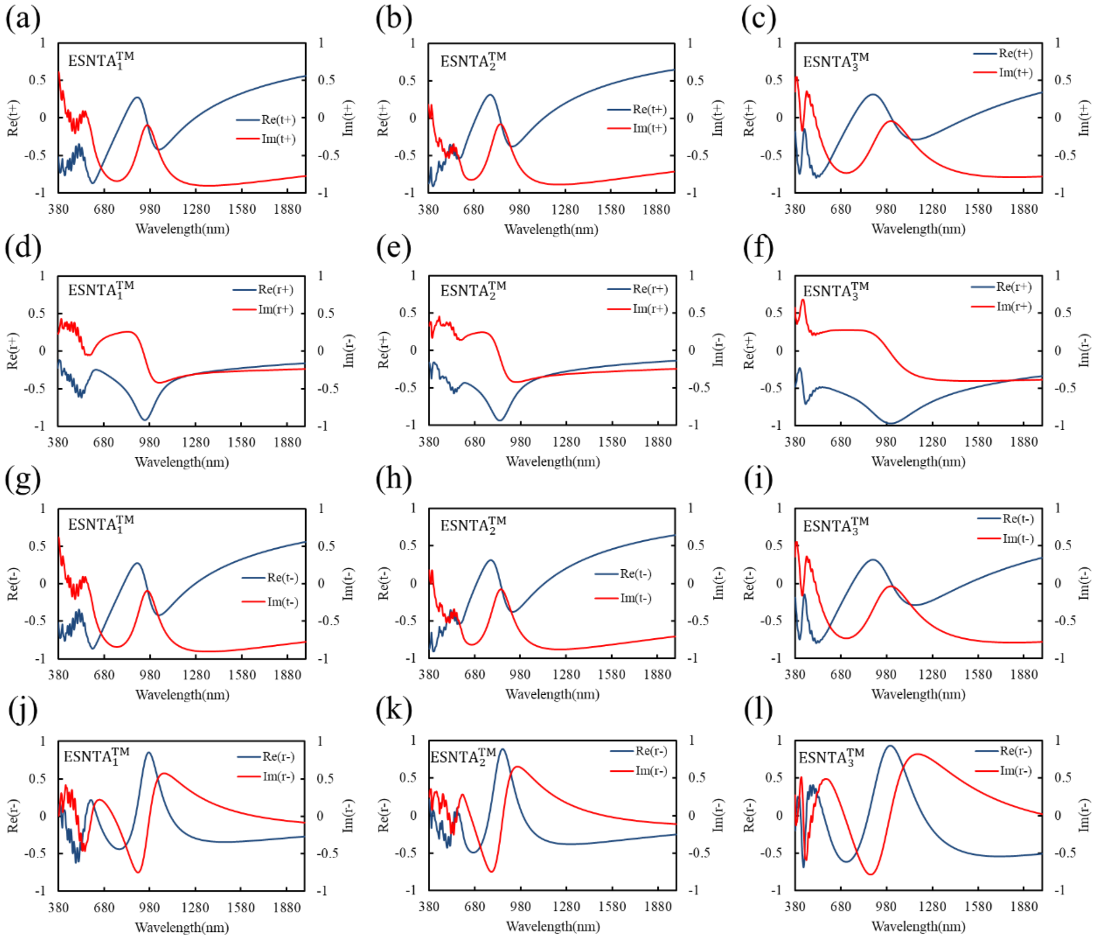

Supplementary Figure 4. Retrieved positive direction (a)-(c) transmission coefficients, (d)-(f) reflection coefficients and negative direction (g)-(i)) transmission coefficients, (j)-(l) reflection coefficients for ESNTA^{TM}.

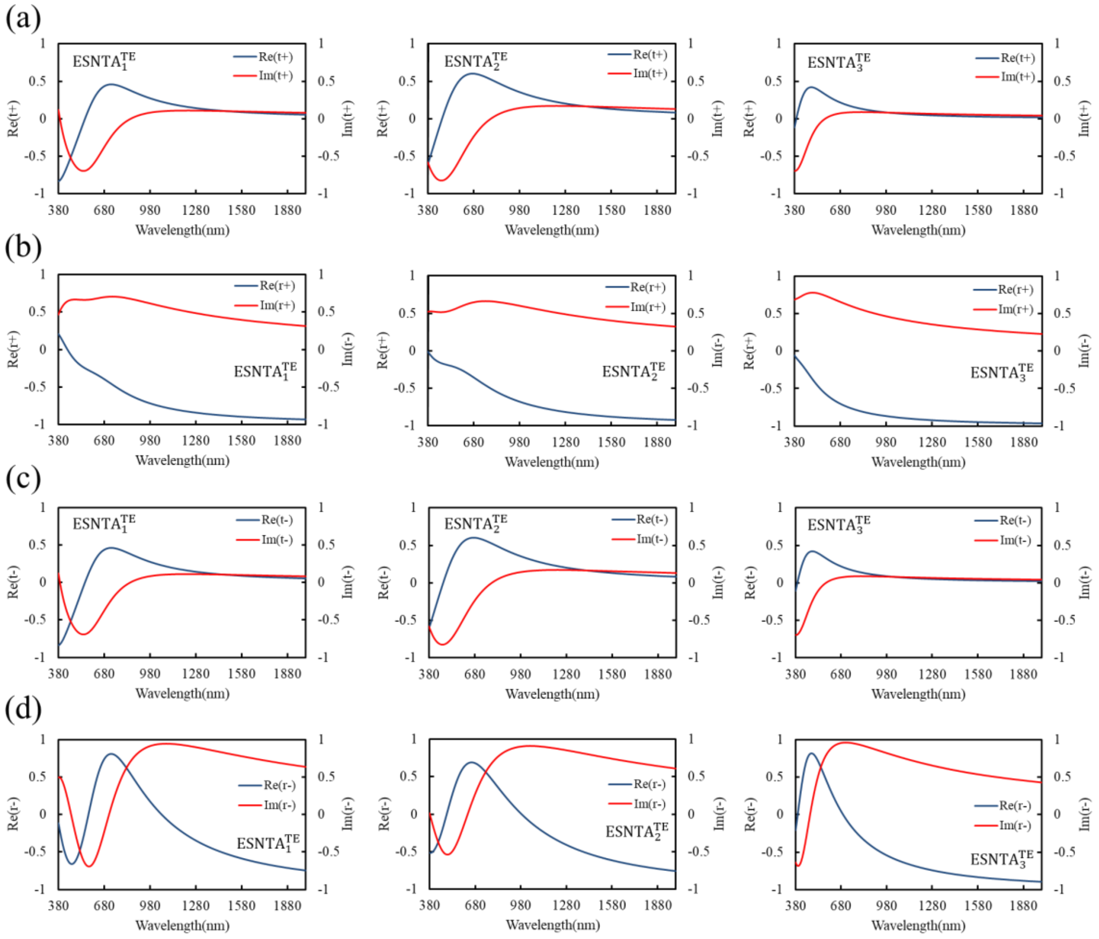

Supplementary Figure 5. Retrieved positive direction (a)-(c) transmission coefficients, (d)-(f) reflection coefficients and negative direction (g)-(i)) transmission coefficients, (j)-(l) reflection coefficients for ESNTA^{TE}.
